# Improved Methods for Fourier-Based Microwave Imaging

**DOI:** 10.3390/s23229250

**Published:** 2023-11-17

**Authors:** Yuri Alvarez López, Fernando Las-Heras Andrés

**Affiliations:** Area of Signal Theory and Communications, Department of Electrical Engineering, Universidad de Oviedo, Edificio Polivalente, Mod. 8, Campus Universitario de Gijón, 33203 Gijón, Spain; flasheras@uniovi.es

**Keywords:** Fourier-based imaging, microwave imaging, plane wave spectrum (PWS), antenna measurement, delay-and-sum (DAS)

## Abstract

Fourier-based imaging has been widely adopted for microwave imaging thanks to its efficiency in terms of computational complexity without compromising image resolution. Together with other backpropagation imaging algorithms like delay-and-sum (DAS), they are based on a far-field approach to the electromagnetic expression relating to fields and sources. To improve the accuracy of these techniques, this contribution presents a modified version of the well-known Fourier-based algorithm by taking into account the field radiated by the Tx/Rx antennas of the microwave imaging system. The impact on the imaged targets is discussed, providing a quantitative and qualitative analysis. The performance of the proposed method for subsampled microwave imaging scenarios is compared against other well-known aliasing mitigation methods.

## 1. Introduction

Microwave imaging systems are able to provide high-resolution images of the targeted scene. They are of special interest in non-destructive testing (NDT) applications [1], e.g., security screening systems for concealed weapon detection [2,3,4], or biomedical applications [5]. Different kinds of microwave imaging algorithms have been developed; some of them, like the backpropagation delay-and-sum (DAS) [6] or Fourier-based algorithms [7,8] are widely used thanks to their efficiency in terms of computational cost and simplicity with respect to those based on full-wave Equations (inverse fast multipole method [9], subspace-based optimization method [10], or gradient-based optimization [11], among others). In these algorithms, the measurements of the field scattered by the targets on the imaging domain are coherently processed to form the microwave image.

DAS and Fourier-based imaging algorithms are founded on a far-field approach of the electromagnetic field expressions relating the radiated fields with their sources, and considering the transmitting (Tx) and receiving (Rx) antennas of the microwave imaging system to be point sources. Although this approach is valid for the majority of the imaging scenarios, in the case of near-field (NF) imaging, the actual field radiated by the Tx/Rx antennas of the imaging system has a certain impact, as described in [1,12]. For example, the Tx/Rx antennas introduce an additional phase shift, resulting in an offset between the true position of the targets and the imaged ones. In [13], the phase error introduced by the antennas of Multiple Input-Multiple Output Synthetic Aperture Radar (MIMO-SAR) systems is analyzed, proposing a time-domain calibration technique to correct it. Another possibility is the use of accurate characterization of the Tx/Rx antennas of the SAR system [12].

Several techniques have been developed in recent years to extend the application of DAS and Fourier-based algorithms to NF imaging scenarios. In [1,14,15], the radiation pattern of the Tx/Rx antenna is introduced in the imaging algorithm to assess its impact on the imaging results. In [16], the DAS algorithm is modified to consider the complex field (both amplitude and phase) radiated by the Tx/Rx antennas of the microwave imaging system. This is accomplished by means of the sources reconstruction method (SRM), which allows obtaining an accurate model of the field radiated by the Tx/Rx antennas [17]. As illustrated in [12], the consideration of not only the amplitude but also the phase of the field radiated by the Tx/Rx antennas can improve the imaging results.

An additional advantage of introducing the field radiated by the Tx/Rx antennas in the imaging algorithm is that it allows for relaxing the sampling rate requirements. This is of particular interest for MIMO-SAR imaging systems [4,18,19,20], as it allows for increasing the spacing between measurements beyond the Nyquist sampling rate (λ/4 for monostatic systems). It also overcomes some of the limitations of compressed-sensing-based sub-Nyquist imaging [8,21], like the high computational cost or the need for a non-uniform sampling of the acquisition domain. An extensive study of the impact of the consideration of the field radiated by the Tx/Rx antennas has been conducted in [16] for the DAS algorithm. Another effective way to minimize the target replicas in the microwave images is achieved by introducing a weighting function in the spectral domain [1,22]. In [23], the aliasing in millimeter-wave imaging systems is studied in the spectral domain (or *k*-space), proposing a mitigation technique in [24] by means of the use of non-uniform Tx and Rx arrays.

Following the idea presented in [16] for the DAS algorithm, this contribution introduces a modified Fourier-based imaging algorithm for monostatic architectures [2] that considers the field radiated by the Tx/Rx antennas. The main novelties of this contribution are listed below:

1. A modified Fourier-based imaging algorithm that takes into account the field radiated by the Tx/Rx antenna, improving the imaging results.
2. An improvement of the computational complexity with respect to existing methods that also include the field radiated by the Tx/Rx antenna (e.g., the modified DAS presented in [12]). The reason is that the introduction of the field radiated by the Tx/Rx antennas is performed in the spectral domain. Thus, it only requires the calculation of the plane wave spectrum (PWS) of the aperture field of the Tx and Rx antennas of the imaging system which is faster than calculating the field radiated in the imaging domain. The analysis of the computational complexity is included in this contribution.
3. The proposed method preserves the advantages of the modified DAS presented in [16]. For example, the capability of imaging the targets at the actual position without the need of a calibration stage, and/or the capability of working with subsampled arrays. A comparison with other Fourier-based imaging methods capable of dealing with subsampled acquisition domains is conducted in this contribution.

For the sake of simplicity, the formulation is developed and validated for a monostatic imaging architecture. Nevertheless, it can be adapted for multistatic radar systems as well [25,26].

## 2. Materials and Methods

### 2.1. Fourier-Based Microwave Imaging

**Figure 1:**
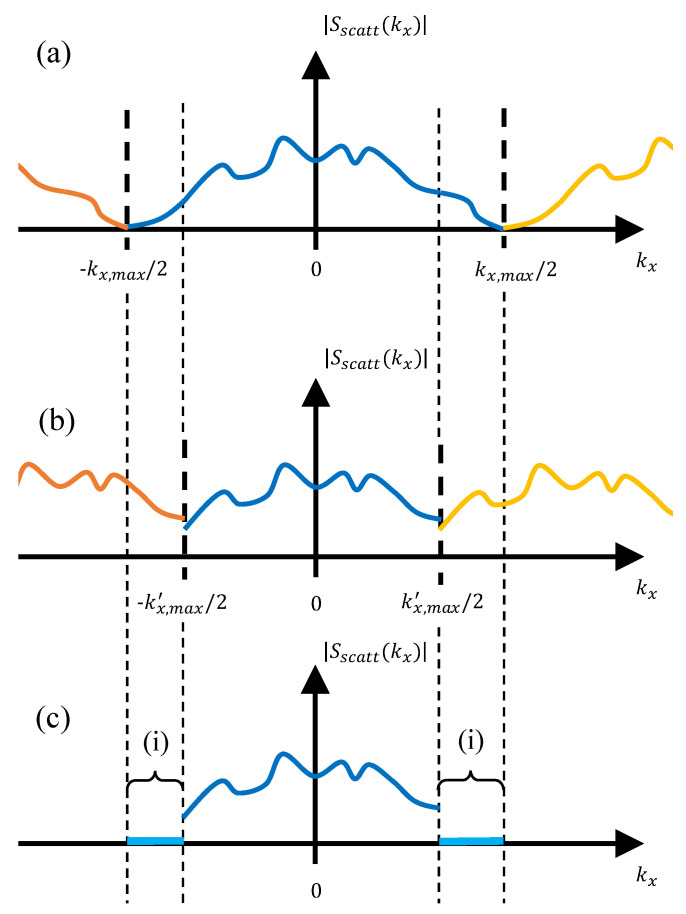
Representation of the PWS of the scattered field (dark blue line: original PWS, orange and yellow lines: PWS replicas). (**a**) Acquisition domain sampling rate: $\delta_{x}$. (**b**) Acquisition domain sampling rate: $\delta_{x}^{\prime }>\delta_{x}$. (**c**) Zero padding of the part of the spectrum denoted by (i) to achieve the same spatial resolution as in Figure 1a.

Let us consider a monostatic microwave imaging system consisting of a planar observation or acquisition domain of size $L_{x}\times L_{y}$ and placed at *z* = 0 m. This acquisition domain is sampled every $\delta_{x}$ and $\delta_{y}$, so that the extension of the plane wave spectrum of the acquired scattered field, $k_{x},k_{y},$ is $k_{x,max}$, $k_{y,max}$ (as depicted in Figure 1a for the $k_{x}$ axis), with $k_{x,y,max}=2\pi /\delta_{x,y}$. The reflectivity in the imaging domain, $\rho (x_{i},y_{i},z_{i})$ (the notation $x_{i},y_{i},z_{i}$ is used to refer to the whole set of voxels of the imaging domain), is given by (1) [2]:

(1) $$\begin{array}{c} \rho \left(x_{i},y_{i},z_{i}\right)=FT_{3D}^{-1}\left\{Interp^{\left(k\to k_{z}\right)}\left\{FT_{2D}\left\{E_{scatt}\left(x,y,\omega \right)\right\}e^{+jk_{z}z_{0}}\right\}\right\}, \end{array}$$

where $E_{scatt}\left(x,y,\omega \right)$ denotes the scattered field acquired in the observation domain, with ω=2πf. $FT_{2D}$ denotes the two-dimensional (2D) Fourier transform from x,y to $k_{x},k_{y}$, that is, $S_{scatt}\left(k_{x},k_{y},\omega \right)=FT_{2D}\left\{E_{scatt}\left(x,y,\omega \right)\right\}$. In the exponential term $e^{+jk_{z}z_{0}}$, $z_{0}$ is the distance (along *z*-axis) between the observation domain, (x,y,0), and the center of the imaging domain, $\left(x_{i},y_{i},z_{i}\right)$, and $k_{z}$ is defined in (2) [2], with k=ω/c.

(2) $$k_{z}=\sqrt{4k^{2}-k_{x}^{2}-k_{y}^{2}}$$

From (2), it is deduced that the relationship between ω (or *k*) and $k_{z}$ is nonlinear, which requires the mapping of the PWS of the acquired scattered field, $S_{scatt}\left(k_{x},k_{y},\omega \right)$, to $S_{scatt}\left(k_{x},k_{y},k_{z}\right)$ (denoted in (1) by $Interp^{\left(k\to k_{z}\right)}$). This step is commonly known as Stolt interpolationn [2,27] and it might have a significant impact on the computational cost depending on the interpolation method chosen. Some authors have proposed different interpolation-free strategies, mainly based on conducting 2D single-frequency imaging at different ranges, then combining coherently the resulting images (range stacking) [8,28].

Finally, $FT_{3D}^{-1}$ denotes the three-dimensional (3D) inverse Fourier transform from $k_{x},k_{y},k_{z}$ to $x_{i},y_{i},z_{i}$: $\rho \left(x_{i},y_{i},z_{i}\right)=FT_{3D}^{-1}\left\{S_{scatt}\left(k_{x},k_{y},k_{z}\right)\right\}$.

**Figure 2:**
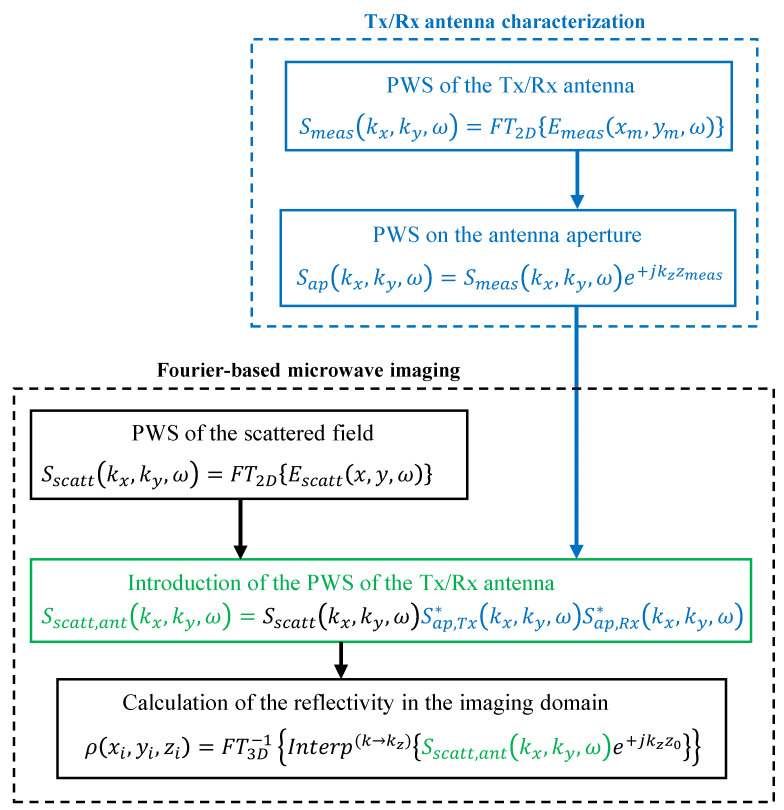
Flowchart of the monostatic Fourier-based microwave imaging method when the field radiated by the Tx/Rx antennas is introduced.

The flowchart of the Fourier-based microwave imaging technique is depicted in black color in Figure 2.

In the case of monostatic imaging systems, the field scattered by the targets must be sampled every λ/4 at the center frequency of the working frequency band (i.e., $\delta_{x,y}\leq \lambda /4$) to avoid aliasing in the PWS, although in practice, this condition can be relaxed to $\delta_{x,y}\leq \lambda /2$ [2,3].

If the scattered field is sampled with a coarser sampling rate, $\delta_{x,y}^{\prime }>\delta_{x,y}$, then $k_{x,y,max}^{\prime }<k_{x,y,max}$, as depicted in Figure 1b, resulting in a loss of spatial resolution in the imaging domain, aside from potential aliasing issues depending on the spectral bandwidth of the scattered field. To address this, a well-known technique is to zero padding the spectral domain from $k_{x,y,max}^{\prime }$ to $k_{x,y,max}$ (Figure 1c) so that the resolution in the spatial domain becomes again $\delta_{x,y}$. Nevertheless, this technique is valid when the spectral bandwidth of the scattered field is less than $k_{x,y,max}^{\prime }$. Otherwise, zero padding may result in a discontinuity of the PWS which can affect the recovered microwave image. Another method to address the issue of subsampled acquisition domains was presented in [25] for multistatic imaging systems. This method is based on the replication of the PWS beyond $\pm k_{x,y,max}^{\prime }/2$ (which results in the same spectrum as shown in Figure 1b); then, a weighting function is applied to filter these replicas out.

**Figure 3:**
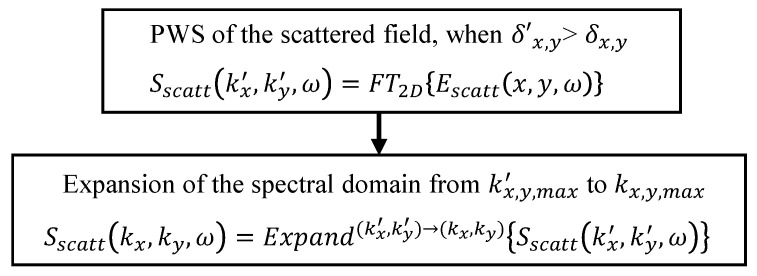
Expansion of the domain of the PWS when the sampling rate of the scattered field is such that $\delta_{x,y}^{\prime }>\delta_{x,y}$.

The step corresponding to the expansion of the PWS of the scattered field is shown in Figure 3, and it is denoted by $Expand^{\left(k_{x}^{\prime },k_{y}^{\prime }\right)\to \left(k_{x},k_{y}\right)}$. This step is conducted right after the 2D Fourier transform of the scattered field from (x,y) to ($k_{x},k_{y}$).

### 2.2. Introduction of the Field Radiated by the Tx/Rx Antennas

The modification of the Fourier-based imaging algorithm outlined in Section 2.1 to introduce the field radiated by the Tx/Rx antennas is described in Figure 2. For the sake of simplicity, a monostatic imaging system with the same Tx and Rx antenna will be considered.

First, the Tx/Rx antenna radiation is characterized, e.g., by measuring its radiated field, $E_{meas}\left(x_{m},y_{m},\omega \right)$ (the notation $x_{m},y_{m}$ denotes the whole set of points where the field radiated by the Tx/Rx antenna was acquired). If the Tx/Rx antenna is directive enough, a planar measurement domain, located at a distance $z_{meas}$ from the Tx/Rx antenna aperture plane, can be used without introducing a significant measurement truncation error [29]. The next step is the calculation of the PWS of the field radiated by the Tx/Rx antenna on its aperture, $S_{ap}\left(k_{x},k_{y},\omega \right)$. This is a well-known technique in the field of antenna measurement that can be implemented by means of a backward transformation from the measurement domain to the aperture plane of the PWS of the antenna, $S_{meas}\left(k_{x},k_{y},\omega \right)$ [30]. This backpropagation is denoted in Figure 2 by $S_{ap}\left(k_{x},k_{y},\omega \right)=S_{meas}\left(k_{x},k_{y},\omega \right)e^{+jk_{z}z_{meas}}$. The steps concerning the measurement and retrieval of the PWS of the Tx/Rx antenna in the aperture plane are highlighted in blue color in Figure 2 (box entitled “Tx/Rx antenna characterization”).

Next, the introduction of the field radiated by the Tx/Rx antenna in the Fourier-based imaging algorithm is achieved by multiplying the PWS of the latter, $S_{ap}^{*}\left(k_{x},k_{y},\omega \right)$, by the PWS of the acquired scattered field, $S_{scatt}\left(k_{x},k_{y},\omega \right)$ (Figure 2, step highlighted in green color). If the Tx and Rx antennas are the same (or a single Tx/Rx antenna is used), then $S_{ap,Tx}^{*}\left(k_{x},k_{y},\omega \right)=S_{ap,Rx}^{*}\left(k_{x},k_{y},\omega \right)$. Note that this step is the same as Equation (8) of the modified DAS in [16], where the actual field radiated by the Tx/Rx antenna is considered instead of the far-field approach of the phase term used in conventional DAS. The difference with the modified DAS is that, in the case of the modified Fourier-based imaging algorithm, the introduction of the actual field radiated by the Tx/Rx antenna is conducted in the spectral domain (or *k*-space), that is, before backpropagating the PWS of the scattered field to the imaging domain (which, in the spectral domain, corresponds to a phase shift, $e^{+jk_{z}z_{0}}$).

**Figure 4:**
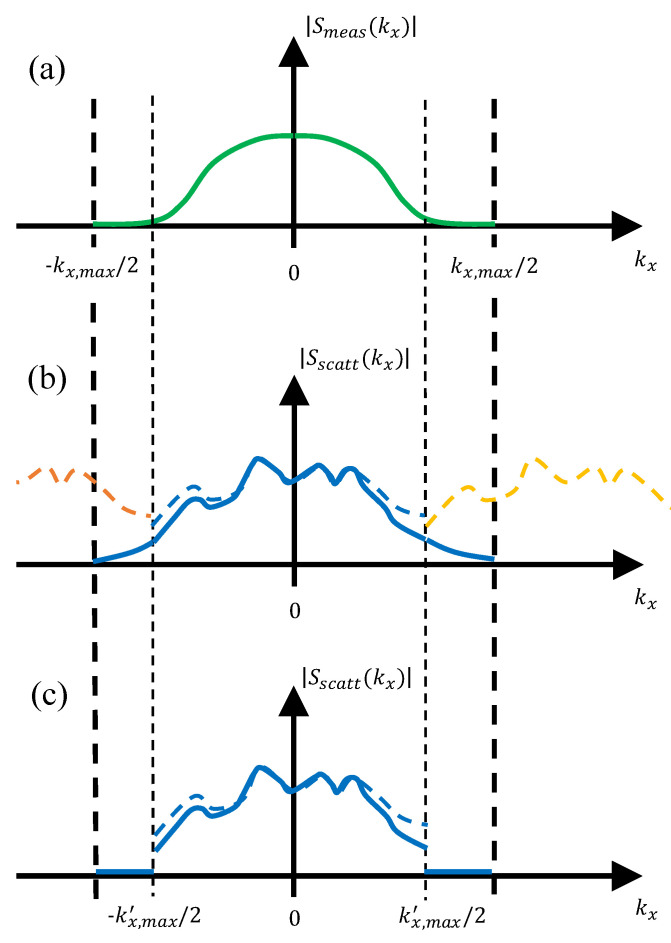
(**a**) Representation of the PWS of the field radiated by the Tx/Rx antenna. (**b**,**c**) Effect of the PWS of the Tx/Rx antenna in the PWS of the scattered field (dashed lines: before introducing the PWS. Solid lines: after introducing the PWS). (**b**) Replication of the PWS of the scattered field. (**c**) Zero padding of the PWS of the scattered field.

The effect of the introduction of the field radiated by the Tx/Rx antenna in the PWS of the scattered field is sketched in Figure 4. The amplitude of the PWS of the field radiated by the Tx/Rx antenna is plotted in Figure 4a. The resulting PWS of the scattered field is shown in Figure 4b when the PWS of the scattered field is replicated (method described in [25]), and in Figure 4c when zero padding is conducted.

As depicted in Figure 2, the PWS of the Tx/Rx antenna is introduced before conducting the backward transformation to the imaging domain ($e^{+jk_{z}z_{0}}$ phase shift) and the Stolt interpolation. That means that the methodology presented in this contribution can be easily incorporated into Stolt interpolation-free Fourier-imaging techniques [8,28].

### 2.3. Analysis of the Computational Complexity

As mentioned in Section 1, the main advantage of Fourier-based imaging techniques over other imaging algorithms like DAS is their efficiency in terms of computational complexity.

The computational cost of microwave imaging algorithms depends on the number of positions where the scattered field is acquired ($N_{scatt}$), and the number of voxels where the reflectivity is reconstructed ($N_{\rho }$). In the case of DAS, this computational cost is $O(N_{scatt}N_{\rho })$. Without loss of generality, it can be assumed $N_{scatt}\approx N_{\rho }$, and thus the computational cost becomes $O(N_{scatt}^{2})$. Fourier-based imaging techniques take advantage of the Fast Fourier Transform (FFT) to lower the computational cost to $O(N_{scatt}log_{2}\left(N_{scatt}\right))$ [31].

When the field radiated by the Tx/Rx antenna is incorporated in the DAS algorithm, the overall calculation time is increased $N_{ap}$ times, where $N_{ap}$ is the number of discrete points where the equivalent currents that characterize the Tx/Rx antenna are calculated. The reason is that, for every measurement, the field radiated by the Tx/Rx antenna is calculated in all the voxels of the imaging domain. If $N_{ap}\ll N_{scatt}$, then the overall computational complexity still remains $O(N_{scatt}^{2})$ [12].

The modified Fourier-based imaging algorithm uses the PWS of the field radiated by the Tx/Rx antenna. The computational cost of computing the PWS is $O(N_{meas,Tx/Rx}log_{2}\left(N_{meas,Tx/Rx}\right))$, where $N_{meas,Tx/Rx}$ is the number of measurements of the field radiated by the Tx/Rx antenna. Next, as explained in Section 2.2, the PWS of the scattered field and the PWS of the field radiated by the Tx/Rx antenna are multiplied, and then, the reflectivity is recovered by computing an inverse Fourier transform. Consequently, the computational complexity of the modified Fourier-based microwave imaging is the sum of $O(N_{meas,Tx/Rx}log_{2}\left(N_{meas,Tx/Rx}\right))$ and $O(N_{scatt}log_{2}\left(N_{scatt}\right))$. If $N_{scatt}\approx N_{meas,Tx/Rx}$, then the overall complexity is $O(N_{scatt}log_{2}\left(N_{scatt}\right))$.

## 3. Validation with Simulations

**Figure 5:**
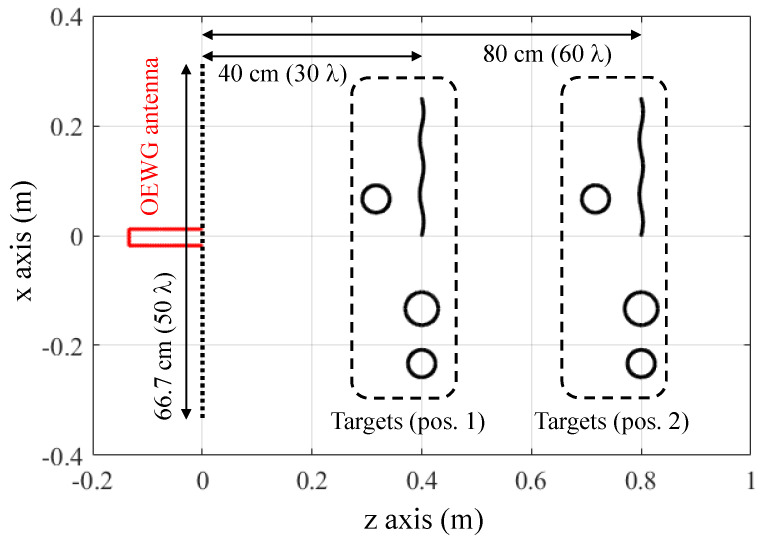
Scheme of the imaging setup analyzed in Section 3.

The proposed method is assessed by means of a two-dimensional (2D) simulation-based example. For comparison purposes, the same simulation scenario presented in Section III.C of [12] is considered, where the Tx/Rx antenna is an Open-Ended Waveguide (OEWG). The working frequency band ranges from 21 to 24 GHz frequency band, being sampled every 150 MHz. The choice of this frequency band is to point out the effects introduced by the field radiated by the OEWG in the recovered microwave images. The monostatic acquisition domain has a 66.7 cm span in the cross-range or *x*-axis, sampled every 0.38 λ at the center frequency of 22.5 GHz. The placement and the size of the targets and the geometry of this 2D scenario are depicted in Figure 5. A full-wave 2D method-of-moments has been used to model the forward scattering problem so that the electromagnetic model of the OEWG antenna can be included in the simulation. In order to assess the impact of the distance between the OEWG antenna and the imaging domain (i.e., where the targets are placed), two different distances were considered (Figure 5).

**Figure 6:**
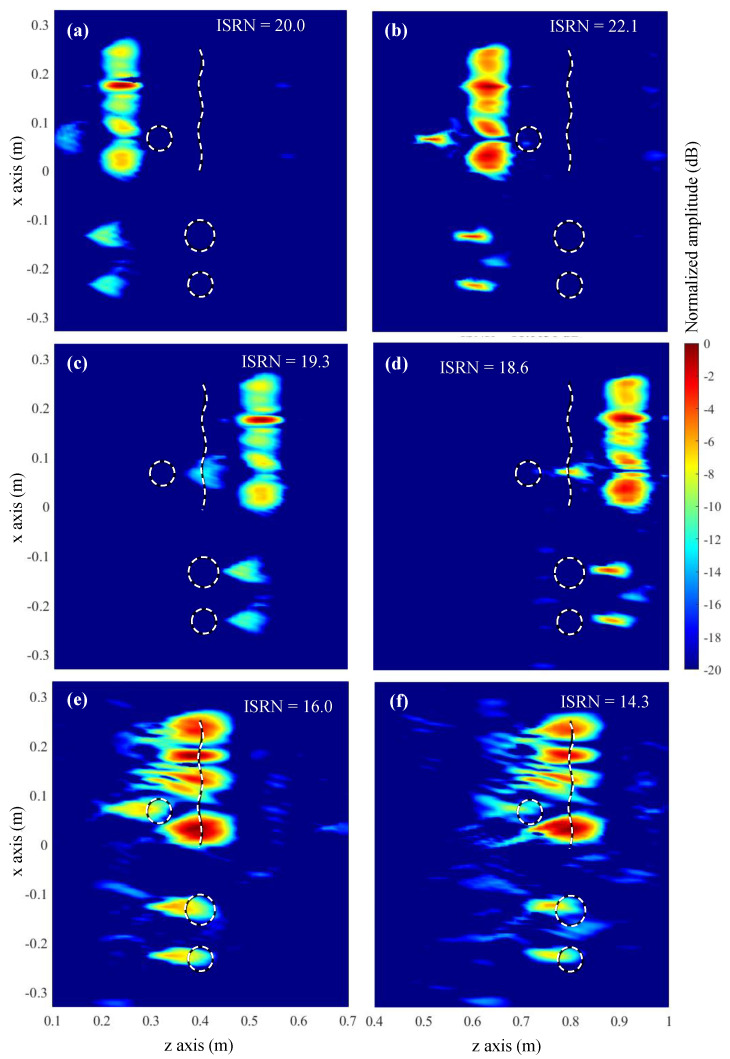
Microwave imaging results of the simulation-based example of Section 3. The acquisition domain is located at *z* = 0 m. (**a**) DAS algorithm, targets 40 cm away from the acquisition domain. (**b**) DAS algorithm, targets 80 cm away. (**c**) Conventional Fourier-based imaging algorithm, targets 40 cm away. (**d**) Fourier-based imaging algorithm, targets 80 cm away. (**e**) Modified Fourier-based imaging algorithm considering the field radiated by the OEWG antenna, targets 40 cm away. (**f**) Modified Fourier-based imaging algorithm considering the field radiated by the OEWG antenna, targets 80 cm away. The white dashed lines indicate the profile of the four metallic targets.

Imaging results are plotted in Figure 6. DAS results without considering the field radiated by the OEWG antenna are depicted in Figure 6a,b for reference purposes. It is observed that, when the targets are 40 cm away from the acquisition domain (Figure 6a), the circular target placed in front of the bent metallic plate is not well imaged. However, for the distance of 80 cm (Figure 6b), the four targets can be identified. Reflectivity images produced by the conventional Fourier-based imaging algorithm, Figure 6c,d, also exhibit the main distinctive features as those obtained with DAS. The explanation is that both DAS and Fourier-based imaging algorithms make use of a far-field approach of the phase term [12]. This approach also results in a shifting in the position of the targets with respect to their actual position, as the additional phase shift introduced by the Tx/Rx antennas is not considered.

Next, the modified Fourier-based imaging algorithm that takes into account the field radiated by the OEWG antenna is applied to process the field scattered by the targets. Results are plotted in Figure 6e,f. As observed for the DAS in [12], the four targets can be identified at both distances (40 cm and 80 cm), at the expense of a lower signal-to-clutter ratio. The latter is quantitatively assessed by means of the Image Signal-to-Noise Ratio (ISNR), defined in Equation (15) of [32]. In this example, when the field radiated by the OEWG antenna is considered, the ISNR decreases.

**Figure 7:**
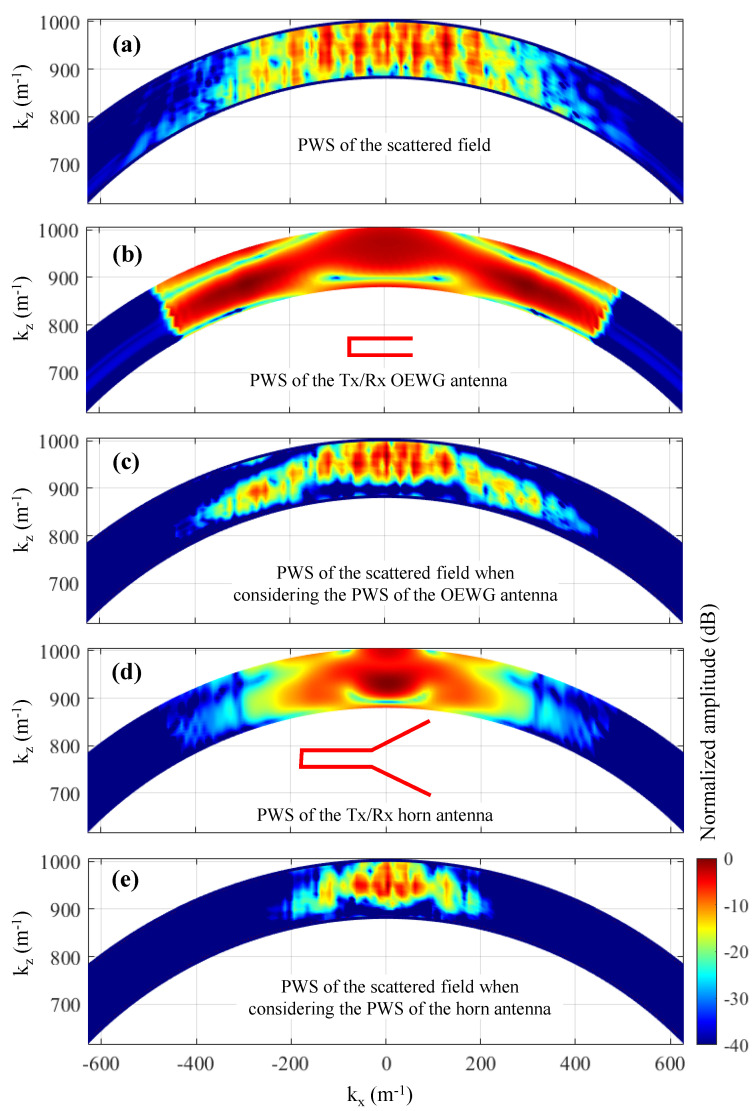
(**a**) PWS of the scattered field of the example of Section 3. (**b**) PWS of the field on the aperture plane of the OEWG antenna. (**c**) PWS of the scattered field after multiplying it by the PWS of the OEWG antenna. (**d**) PWS of the field on the aperture plane of the horn antenna. (**e**) PWS of the scattered field after multiplying it by the PWS of the horn antenna.

The PWS of the acquired scattered field and the OEWG antenna are depicted in Figure 7a,b, respectively, together with the result of multiplying both PWS, Figure 7c. In the latter plot, the tapering effect in the PWS of the scattered field introduced by the PWS of the OEWG can be observed.

### 3.1. Resolution Analysis

**Figure 8:**
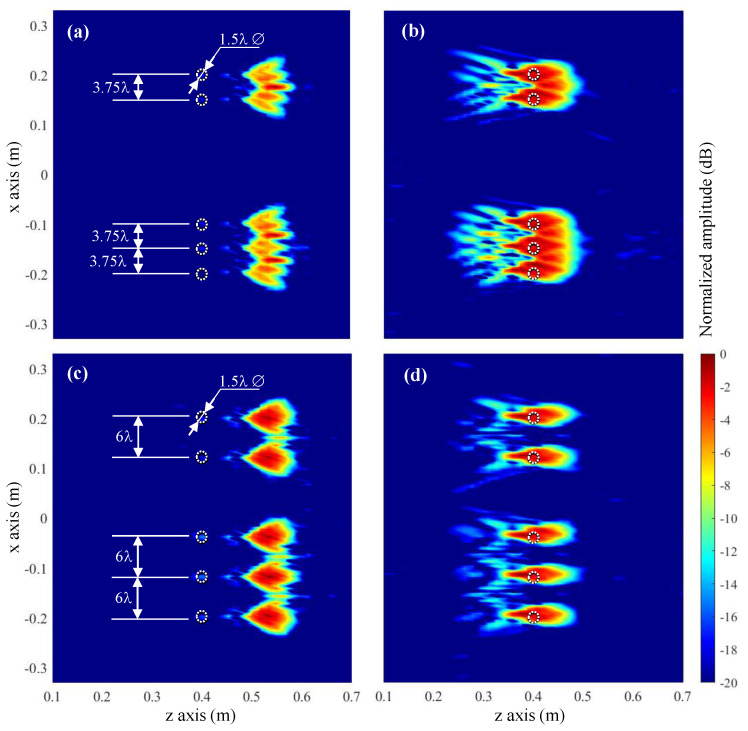
Resolution analysis in which 1.5 λ-diameter circular targets, spaced 3.75 λ (**a**,**b**) and 6 λ (**c**,**d**) were considered. (**a**,**c**) Fourier-based imaging technique. (**b**,**d**) Fourier-based imaging technique considering the field radiated by the Tx/Rx OEWG antenna.

In this subsection, the resolution achieved with conventional and modified Fourier-based imaging algorithms will be tested by considering small circular metallic targets with different spacing, depicted in Figure 8a,c. This analysis will be conducted for the case where the targets are 40 cm away from the acquisition domain, as shown in Figure 6c,e; this is the case where the introduction of the field radiated by the Tx/Rx antenna improves the imaging results with respect to conventional Fourier-based imaging.

Results are plotted in Figure 8, where it can be observed that the circular targets cannot be distinguished when spaced less than 4 λ (Figure 8a,b). When the spacing is wider, 6 λ, all the targets can be identified. The consideration of the field radiated by the Tx/Rx OEWG antenna provides better contrast (Figure 8d) than the conventional Fourier-based imaging method (Figure 8c). As in the previous example, only when the field radiated by the OEWG antenna is considered, the circular scatterers are imaged at the right position.

### 3.2. Comparison of Different Tx/Rx Antennas

This section analyzes the imaging results when different Tx/Rx antennas are considered. Results achieved with the OEWG antenna of previous examples will be compared against those obtained with a horn antenna, which is more directive than the OEWG. For this case, the forward scattering problem has been computed considering the horn antenna model (as in the example in which the Tx/Rx antenna was the OEWG).

The impact of the kind of Tx/Rx antenna in the resolution of the microwave images depends on the spectral bandwidth of the antenna, which is related to its directivity. Thus, directive Tx/Rx antennas (e.g., horn antennas, see Figure 7d) will exhibit less spectral bandwidth than low directive antennas (e.g., OEWG antennas, Figure 7b). If the spectral bandwidth of the scattered field is wider than the spectral bandwidth of the Tx/Rx antenna, then the PWS of the scattered field will be filtered out by the PWS of the Tx/Rx antenna, resulting in a smoothing of the microwave image.

**Figure 9:**
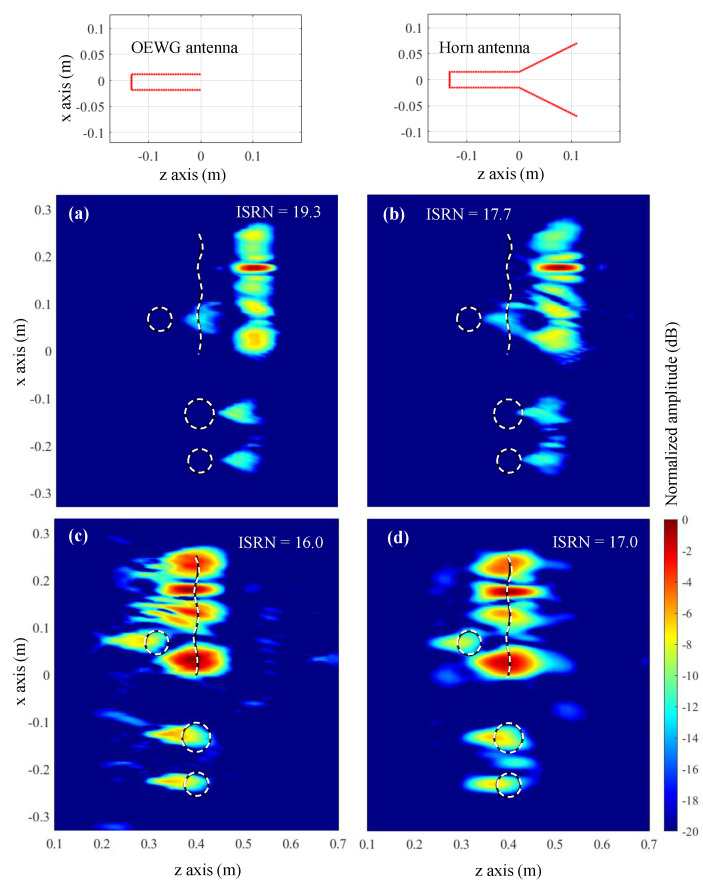
Comparison of the imaging results for different Tx/Rx antennas. (**a**) OEWG antenna, Fourier-based imaging technique. (**b**) Horn antenna, Fourier-based imaging technique. (**c**) OEWG antenna, Fourier-based imaging technique when the field radiated by the Tx/Rx antenna is introduced. (**d**) Horn antenna, Fourier-based imaging technique when the field radiated by the Tx/Rx antenna is introduced.

Imaging results when the simulated scattered field is acquired using the OEWG antenna and the horn antenna are depicted in Figure 9. Fourier-based imaging algorithm without and with considering the field radiated by the Tx/Rx antenna are compared as well. It can be observed that, regardless of the kind of antenna used in the acquisition of the scattered field, the major improvement happens when the field radiated by the Tx/Rx antenna is incorporated into the imaging algorithm (Figure 9c,d). Concerning the influence of the kind of antenna, it is noticed that reflectivity images retrieved from the scattered field acquired with the horn antenna (Figure 9b,d) are smoother than those obtained from the scattered field acquired with the OEWG (Figure 9a,c). This is consistent with the explanation given in the previous paragraph.

### 3.3. Subsampled Acquisition Domain

The impact of subsampling is analyzed in this section. The same simulation scenario as in Section 3.2 will be kept, keeping the horn antenna as the Tx/Rx antenna. The reason is that horn antennas are more directive than OEWG antennas and thus they are more effective in reducing aliasing in subsampled acquisition domains (as illustrated in Figure 7 of [16]).

Starting from the sampling rate considered in the previous simulation-based examples, $\delta_{x}$ = 0.38 λ, this rate was increased by a sampling factor of $N_{\delta }$, with $N_{\delta }$ = 2, 3, …until finding a value for which none of the tested Fourier-based imaging algorithms were able to provide a reflectivity image where the targets could be distinguished. For this example, this value was $N_{\delta }$ = 4. Thus, the maximum sampling rate for which the targets could be imaged was $N_{\delta }$ = 3, that is, $\delta_{x}$ = 1.14 λ (within the order of the subsampling rate achieved in the example of Section III.B of [16]).

**Figure 10:**
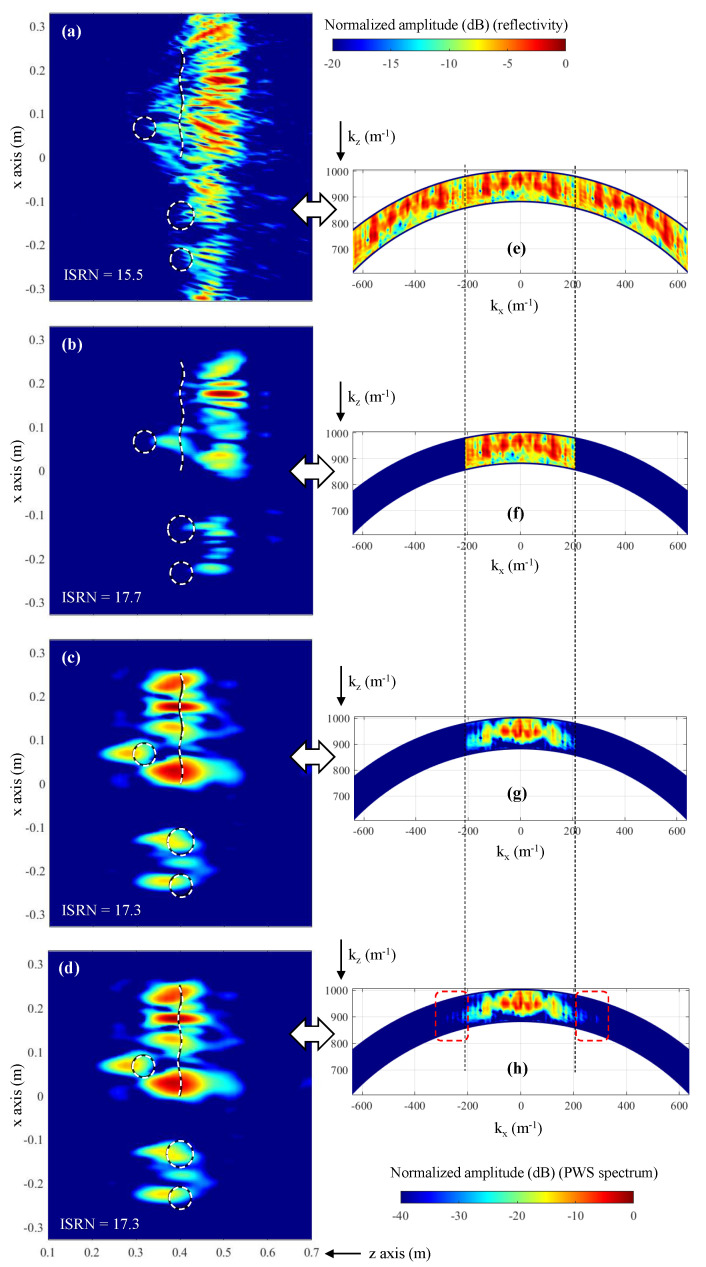
Fourier-based imaging results when the sampling rate of the scattered field is $\delta_{x}$ = 1.14 λ. (**a**) Reflectivity recovered with the conventional Fourier-based imaging. (**b**) Reflectivity recovered with the Fourier-based imaging, using zero padding. (**c**) Reflectivity recovered with the modified Fourier-based imaging algorithm when the spectral domain is filled with zero padding. (**d**) Reflectivity recovered with the modified Fourier-based imaging algorithm, without zero padding. (**e**) PWS of the scattered field. (**f**) PWS of the scattered field, when zero padding is applied. (**g**) PWS of the scattered field, when zero padding is applied, and after multiplying it by the PWS of the horn antenna. (**h**) PWS of the scattered field, without applying zero padding, and after multiplying it by the PWS of the horn antenna. For the plots of the left column, the white dashed lines indicate the profile of the four metallic targets. In PWS plots, the black dashed lines denote the spectral bandwidth (from $-k_{x,max}^{\prime }$ to +$k_{x,max}^{\prime }$) of the subsampled scattered field.

Imaging results for different Fourier-based imaging methods are depicted in Figure 10, together with the corresponding PWS. The reflectivity recovered with the conventional Fourier-based imaging algorithm is depicted in Figure 10a. Due to the aliasing, none of the four targets can be identified. As the acquisition domain is subsampled, the replicas of the spectrum of the scattered field overlap, as observed in Figure 10e.

Depending on the degree of overlapping between the replicas of the spectrum of the scattered field, zero padding can be applied to minimize the impact of aliasing in the recovered reflectivity image. Given the desired spatial resolution of $\delta_{x}$ = 0.38 λ, the spectral bandwidth between $k_{x,max}^{\prime }$ and $k_{x,max}$ is filled with zeros (zero padding), as noticed in Figure 10f (dark blue regions). The recovered reflectivity image is shown in Figure 10b: as in Figure 6c, the targets are shifted with respect to their actual position, and the reflectivity level of the circular targets is 10–15 dB less than the reflectivity level of the bent metallic plate.

The PWS depicted in Figure 10g corresponds to the zero-padded PWS of the scattered field (Figure 10f multiplied by the PWS of the aperture field of the horn antenna (Figure 7d). As shown in Figure 7e, a tapering effect is observed. When the reflectivity is recovered from this PWS, not only are the targets imaged at the actual position, but also the reflectivity levels of the four targets are within a 5 dB margin (as was the case for Figure 6e).

Finally, the PWS depicted in Figure 10e is also multiplied by the PWS of the aperture field of the horn antenna (Figure 7d), resulting in the PWS plotted in Figure 10h. It can be observed that the resulting PWS has almost the same spectral bandwidth as the PWS horn antenna, which is consistent with the fact that the spectral bandwidth of the horn antenna is approximately $k_{x,max}^{\prime }$. Thus, the replicas of the PWS of the scattered field outside the -$\left[k_{x,max}^{\prime }\;k_{x,max}^{\prime }\right]$ interval are attenuated (highlighted with dashed red lines in Figure 10h). The recovered reflectivity is shown in Figure 10d, being almost identical to the one recovered with the modified Fourier-based imaging when zero padding is conducted in the spectral domain.

ISNR values are also provided for the reflectivity images shown in Figure 10a–d. As expected, the lowest value corresponds to Figure 10a, which is the one distorted by aliasing.

## 4. Experimental Validation

**Figure 11:**
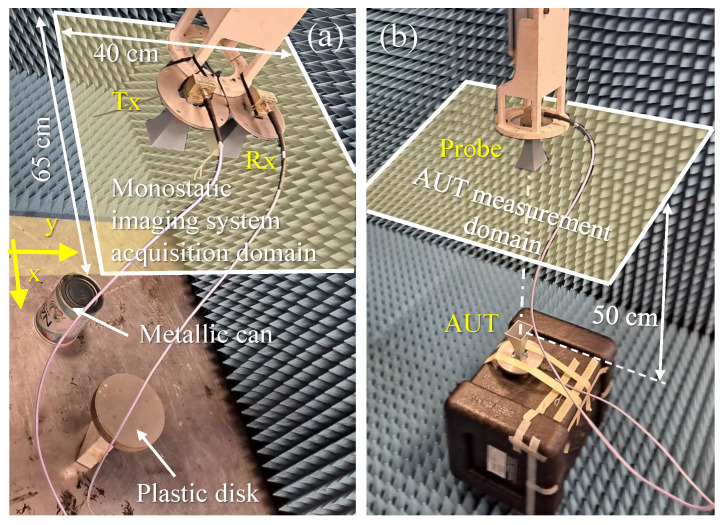
Picture of the measurement setup. (**a**) Microwave imaging setup. (**b**) Antenna characterization setup.

Validation with measurements has been carried out using the facility described in [33]. Figure 11a shows the monostatic microwave imaging setup in the 12–18 GHz frequency band. Two targets were considered: an 18 cm long × 14 cm ⌀ metallic can, and an 18 cm ⌀ and 4 cm thick plastic disk, placed 22 cm above the metallic floor of the facility. The size of the scattered field acquisition domain was $L_{x}\times L_{y}$ = 65 cm × 40 cm, discretized every $\delta_{x,y}$ = 10 mm (λ/2 at the center frequency of 15 GHz), and placed 1 m above the floor of the measurement facility. The Tx and Rx antennas were two horn antennas [34] connected to a Vector Network Analyzer (VNA) using 2 m long cables. To remove the additional phase shift introduced by these cables, the measurement setup was calibrated at the connection between the antennas and the cables using a mechanical Open-Short-Load-Through (OSLT) calibration kit.

The field radiated by the horn antennas was measured in the same 12–18 GHz frequency band, on an acquisition domain discretized also every 10 mm, placed 50 cm above the antenna aperture (Figure 11b), and it was processed as indicated in the flowchart of Figure 2. More precisely, the backward transformation algorithm described in [30] was applied to obtain the PWS on the aperture plane of the field radiated by the horn antenna.

**Figure 12:**
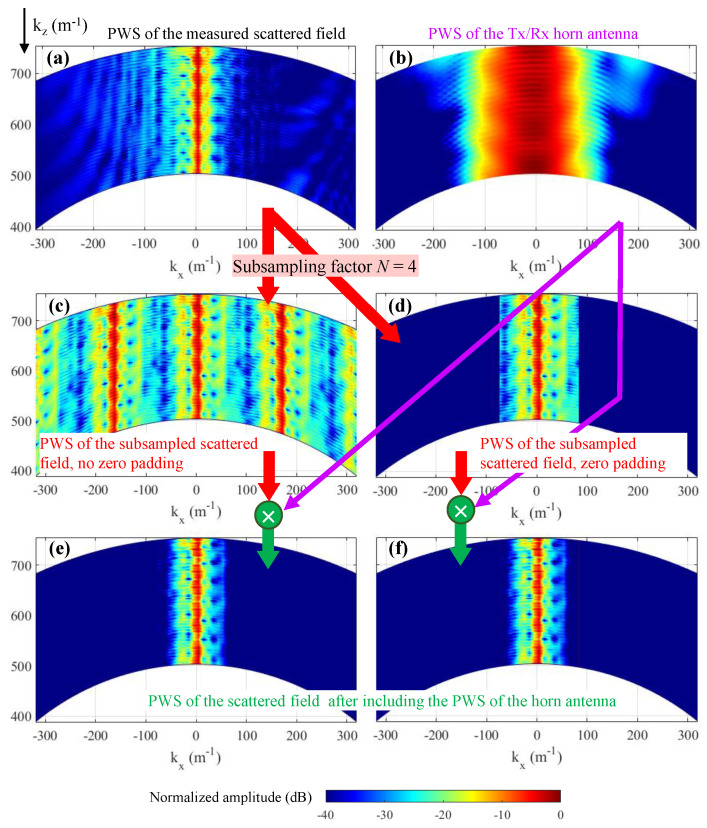
Representation of the PWS for different measurements and methods. (**a**) PWS of the measured scatterd field. (**b**) PWS of the Tx/Rx horn antenna. (**c**) PWS of the subsampled scattered field, no zero padding. (**d**) PWS of the subsampled scattered field, zero padding. (**e**) PWS of the subsampled scattered field (without zero padding) after multiplying it by the PWS of the horn antenna. (**f**) PWS of the subsampled scattered field (with zero padding) after multiplying it by the PWS of the horn antenna.

**Figure 13:**
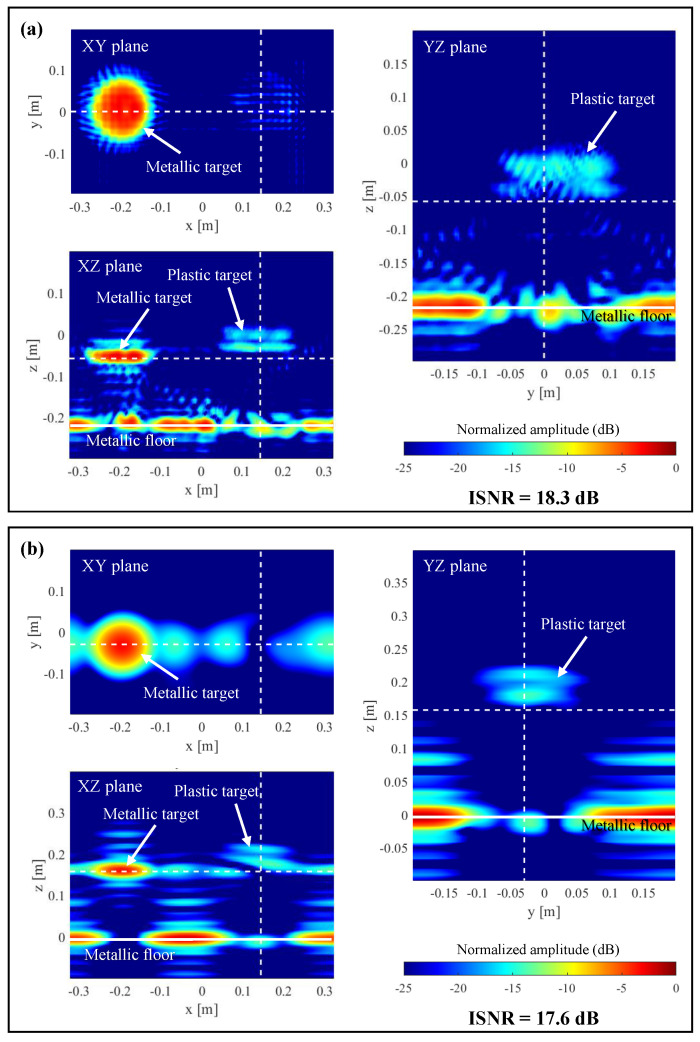
Imaging results for the measurement setup depicted in Figure 11a. XY, XZ, and YZ cuts are centered at the location of the targets. The plane *z* = 0 m corresponds to the actual position of the flat metallic floor. (**a**) Conventional Fourier-based imaging. (**b**) Fourier-based microwave imaging when the PWS of the horn antenna is considered. Dashed white lines on each plane denote the position of the other two planes (e.g., XZ plane and YZ plane in the case of the XY plane representation).

The PWS of the measured scattered field as well as the PWS of the field radiated by the horn antenna on the aperture plane are depicted in Figure 12a,b, respectively. In this example, the spectral bandwidth of the horn antenna is broader than the spectral bandwidth of the scattered field. The reflectivity recovered from the PWS of the scattered field without and with multiplying by the PWS of the horn antenna is plotted in Figure 13. As in the simulation-based examples, the targets are imaged backward with respect to their actual position when the field radiated by the horn antenna is not taken into account. Another effect observed in Figure 13 is that the modified Fourier-based imaging worsens the spatial resolution with respect to conventional Fourier-based imaging. This is due to the taper introduced by the PWS of the horn antenna in the PWS of the scattered field (as previously observed in Figure 7). Despite this, the upper and lower interfaces of the plastic disk can be identified in Figure 13b.

**Figure 14:**
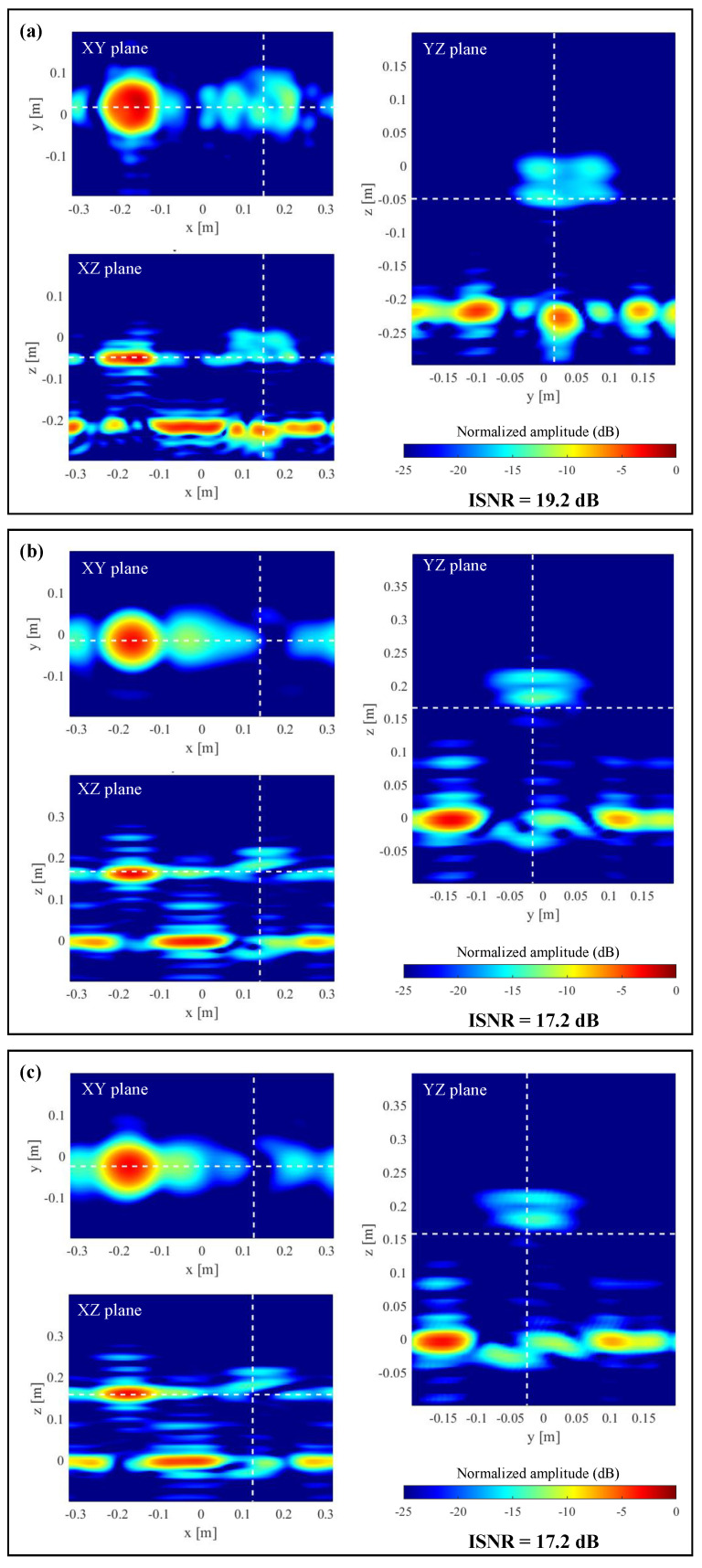
Imaging results for the measurement setup depicted in Figure 11a, when the subsampling rate is $N_{\delta }$ = 4. Reflectivity recovered from the following PWS of the scattered field: (**a**) The PWS is zero-padded. (**b**) Zero-padded PWS times the PWS of the horn antenna. (**c**) Subsampled PWS (no zero-padding) times the PWS of the horn antenna. Dashed white lines on each plane denote the position of the other two planes (e.g., XZ plane and YZ plane in the case of the XY plane representation).

Next, the performance of the modified Fourier-based imaging when the acquisition domain is subsampled is tested. In this example, the maximum subsampling rate achieved was $N_{\delta }$ = 4, that is, $\delta_{x,y}=2\lambda$. The PWS corresponding to the subsampled scattered field is plotted in Figure 12c, where the spectrum replicas are observed. Zero-padded PWS is plotted in Figure 12d, and the recovered reflectivity for this case is shown in Figure 14a. It is noticed that the recovered image worsens with respect to Figure 13a. For example, the lower and upper interfaces of the plastic disk are barely observed.

Finally, the PWS of the subsampled scattered field (Figure 12c,d) is multiplied by the PWS of the horn antenna (Figure 12b). As in the results presented in the simulation-based example of Section 3.3, there is little difference between the PWS of the scattered field due to the tapering introduced by the PWS of the horn antenna. Reflectivity results are plotted in Figure 14b,c: with respect to zero-padding results (Figure 14a), the upper and lower interfaces of the plastic disk are better identified.

The assessment of the computational cost (calculation time and memory consumption) for this 3D microwave imaging is provided in Table 1. A conventional laptop with 16 GB RAM and 6 Intel Core i7 processors was used, and the imaging algorithm was coded using Matlab^TM^ (version: 9.14.0.2337262 (R2023a)).

Additional remarks about the calculation time and memory consumption values shown in Table 1 are given below:

- DAS just requires the calculation of the far-field phase term, whereas the DAS with the field radiated by the Tx/Rx antenna uses the numerical evaluation of the integral equations relating the equivalent currents that characterize the Tx/Rx antenna and the points of the imaging domain [16]. That is the reason why the multiplication of the calculation time of DAS times $N_{ap}$ (for this example, 268 s × 240 = 64,320 s) is smaller than the calculation time of DAS with the field radiated by the Tx/Rx antenna (181,230 s).
- In the case of Fourier-based imaging, the step with the highest memory consumption and calculation time in the implemented code corresponds to the Stolt interpolation [2,27].
- The calculation time and memory consumption depend on how these methods are implemented. For example, DAS implementation is based on a cumulative sum of the reflectivity, where the reflectivity value for each frequency and measurement position is computed and added to the previous values, thus avoiding the need to store large matrices.
- All in all, the calculation time and memory consumption will be influenced by the way these backpropagation algorithms are coded in Matlab^TM^. Thus, the asymptotic study of the computational cost conducted in Section 2.3 is more rigorous than the one presented in this section, as it does not depend on how the methods are implemented and coded.

## 5. Conclusions

This contribution has presented a modification of the Fourier-based imaging algorithm to account for the field radiated by the Tx/Rx antennas of the microwave imaging system. The use of the PWS of the aperture fields of the antennas allows for preserving the efficiency of the Fourier-based technique in terms of computational complexity (especially when compared to DAS), while keeping the improvements associated with the use of the actual field radiated by the Tx/Rx antennas.

The introduction of the field radiated by the Tx/Rx antenna in the Fourier-based microwave imaging algorithm has several advantages that have been shown in the simulation and measurement examples presented in this contribution. First, the modified Fourier-based imaging allows for a better imaging of the targets (Figure 6) than the conventional Fourier-based algorithm. The reason is that the far-field approach of the phase term might be not accurate enough when the targets are close to the acquisition domain. The consideration of the phase of the field radiated by the Tx/Rx antenna allows imaging of the targets at their actual position, as the additional phase shift due to the Tx/Rx antenna can be characterized and incorporated into the imaging technique. Finally, concerning microwave imaging systems that work with subsampled acquisition domains, the modified Fourier-based imaging technique performs as well as the zero-padding method while incorporating the two aforementioned improvements (as illustrated in Figure 10).

The proposed method has been validated for monostatic imaging architectures, but it can be extended to other kinds of imaging architectures (e.g., multistatic ones).

## Figures and Tables

**Table 1:** Calculation time and memory consumption.

Problem Size		
• Number of acquisition points of the scattered field ($N_{scatt}$): 41 × 66 = 2706.		
• Number of measurements of the field radiated by the Tx/Rx antenna ($N_{meas,Tx/Rx}$): 66 × 81 = 5346.		
• Number of equivalent currents used to characterize the Tx/Rx antenna ($N_{ap}$): 240.		
• Points of the imaging domain ($N_{\rho }$): 41 × 66 × 61 = 165,066.		
**Method**	**Calculation time**	**Memory consumption**
DAS [6]	268 s	33 MB ^1^
DAS considering the field radiated by the Tx/Rx antenna [16]	181,230 s (50.3 h)	45 MB ^1^
Fourier-based imaging [7,8]	64 s	264 MB
Fourier-based imaging considering the field radiated by the Tx/Rx antenna (this contribution)	76 s	283 MB

^1^ Cumulative sum used to calculate reflectivity.

## Data Availability

The data presented in this study (Section 4) are openly available in IEEEDataport, reference number (DOI) https://dx.doi.org/10.21227/2dbm-qc30.

## Abbreviations

The following abbreviations are used in this manuscript:

DAS	Delay-and-Sum algorithm.
FFT	Fast Fourier Transform.
ISNR	Image Signal-to-Noise Ratio.
MIMO	Multiple Input Multiple Output (radar system).
NF	(Scattered or radiated) near field.
NDT	Non-destructive testing.
OEWG	Open-ended waveguide.
OSLT	Open-Short-Load-Through calibration kit.
PWS	Plane wave spectrum (of the scattered or radiated field).
Rx	Receiving antenna.
SRM	Sources Reconstruction Method.
SAR	Synthetic Aperture Radar.
3D	Three-dimensional.
Tx	Transmitting antenna.
2D	Two-dimensional.
